# IgG Autoantibodies against β_2_-Glycoprotein I Complexed with a Lipid Ligand Derived from Oxidized Low-Density Lipoprotein are Associated with Arterial Thrombosis in Antiphospholipid Syndrome

**DOI:** 10.1080/10446670310001642113

**Published:** 2003

**Authors:** Daniel Lopez, Kazuko Kobayashi, Joan T. Merrill, E. Matsuura, Luis R. Lopez

**Affiliations:** 1 Corgenix Inc. 12061 Tejon St. Westminster CO 80234 USA; 2 Department of Cell Chemistry Okayama University Graduate School of Medicine and Dentistry 2-5-1 Shikata-cho Okayama 700-8558 Japan; 3 Oklahoma Medical Research Foundation 825 NE 13th St. Oklahoma City OK 73104 USA

## Abstract

We recently reported [*J. Lipid Res. * **42** (2001), 697; **43** (2002), 1486; **44** (2003), 716] that β_2_-glycoprotein I (β_2_GPI) forms complexes with oxidized LDL (oxLDL) and autoantibodies against these complexes are present in patients with SLE and antiphospholipid syndrome (APS). The relationship of β_2_GPI/oxLDL complexes and IgG autoantibodies against β_2_GPI complexed with oxLig-1 (an oxLDL-derived ligand) with clinical manifestations of APS was studied in 150 APS and SLE patients. The β_2_GPI/oxLDL levels of APS patients were similar to those of SLE patients without APS, but they were significantly higher than healthy individuals. There was no difference in the complex levels among the patients with arterial, venous thrombosis, or pregnancy morbidity. IgG anti-β_2_GPI/oxLig-1 levels of APS were significantly higher than those of SLE without APS and healthy individuals. Further, antibody levels of APS patients with arterial thrombosis were significantly higher than those patients with venous thrombosis and pregnancy morbidity. Thus, oxidation of LDL leads the complex formation with β_2_GPI in SLE and APS patients. In contrast, anti-β_2_GPI/oxLig-1 autoantibodies were generated only in APS and were strongly associated with arterial thrombosis. These results suggest that autoantibodies against β_2_GPI/oxLDL complexes are etiologically important in the development of atherosclerosis in APS.


*Clinical & Developmental Immunology*, June–December 2003 Vol. 10 (2–4), pp. 203–211

Taylor & Francis healthsciences

### IgG Autoantibodies against $\beta_2$-Glycoprotein I Complexed with a Lipid Ligand Derived from Oxidized Low-Density Lipoprotein are Associated with Arterial Thrombosis in Antiphospholipid Syndrome

DANIEL LOPEZ[a], KAZUKO KOBAYASHI[b], JOAN T. MERRILL[c], E. MATSUURA[b,*] and LUIS R. LOPEZ[a]

[a]*Corgenix Inc., 12061 Tejon St., Westminster, CO 80234, USA;* [b]*Department of Cell Chemistry, Okayama University Graduate School of Medicine and Dentistry, 2-5-1 Shikata-cho, Okayama 700-8558, Japan;* [c]*Oklahoma Medical Research Foundation, 825 NE 13th St. Oklahoma City, OK 73104, USA*

We recently reported [*J. Lipid Res.* **42** (2001), 697; **43** (2002), 1486; **44** (2003), 716] that $\beta_2$-glycoprotein I ($\beta_2$GPI) forms complexes with oxidized LDL (oxLDL) and autoantibodies against these complexes are present in patients with SLE and antiphospholipid syndrome (APS). The relationship of $\beta_2$GPI/oxLDL complexes and IgG autoantibodies against $\beta_2$GPI complexed with oxLig-1 (an oxLDL-derived ligand) with clinical manifestations of APS was studied in 150 APS and SLE patients. The $\beta_2$GPI/oxLDL levels of APS patients were similar to those of SLE patients without APS, but they were significantly higher than healthy individuals. There was no difference in the complex levels among the patients with arterial, venous thrombosis, or pregnancy morbidity. IgG anti-$\beta_2$GPI/oxLig-1 levels of APS were significantly higher than those of SLE without APS and healthy individuals. Further, antibody levels of APS patients with arterial thrombosis were significantly higher than those patients with venous thrombosis and pregnancy morbidity. Thus, oxidation of LDL leads the complex formation with $\beta_2$GPI in SLE and APS patients. In contrast, anti-$\beta_2$GPI/oxLig-1 autoantibodies were generated only in APS and were strongly associated with arterial thrombosis. These results suggest that autoantibodies against $\beta_2$GPI/oxLDL complexes are etiologically important in the development of atherosclerosis in APS.

*Keywords*: Antiphospholipid antibodies; Antiphospholipid syndrome; Anti-oxidized LDL antibodies; Arterial thrombosis; Atherosclerosis; $\beta_2$-glycoprotein I

### INTRODUCTION

High serum levels of antiphospholipid antibodies have been associated with thromboembolic events of both the arterial and venous vasculature, and with pregnancy morbidity (miscarriages and fetal loss). These features are major criteria for the classification of the antiphospholipid syndrome (APS), a clinical entity that may be present in the context of a systemic autoimmune disorder (secondary APS), or in the absence of an underlying disease (primary APS) (Hughes *et al.*, 1986; Gharavi *et al.*, 1987). Antiphospholipid antibodies, anti-cardiolipin antibodies (aCL) or lupus anticoagulants, are a heterogeneous group of autoantibodies with a possible pathogenic role in the development of the clinical manifestations of APS. These antibodies are characterized by their reactivity to negatively charged phospholipids, phospholipid/protein complexes, and certain proteins presented on suitable surfaces (i.e. activated cell membranes, oxygenated polystyrene) (Matsuura *et al.*, 1994; Roubey, 1994).

Several plasma proteins that participate in coagulation and interact with anionic phospholipids have been described as antiphospholipid cofactors, i.e. $\beta_2$-glycoprotein I ($\beta_2$GPI), prothrombin, and annexin V. These protein cofactors have been shown to be relevant antigenic targets for antiphospholipid antibodies (Matsuura *et al.*, 1990; McNeil *et al.*, 1990). $\beta_2$GPI is a 50 kDa single-chain polypeptide composed of 326 amino acid residues, arranged in 5 homologous repeats known as complement control protein domains. *In vitro*, $\beta_2$GPI binds strongly to anionic molecules, such as negatively charged phospholipids, heparin, and lipoproteins, as well as to activated platelets and apoptotic cell membranes. Further, $\beta_2$GPI has anticoagulant properties, as it has been shown to inhibit the intrinsic coagulation pathway, prothrombinase activity, and ADP-dependent platelet aggregation (Sheng *et al.*, 1998). It has also been reported to interact with

*Corresponding author. Tel.: +81-86-235-7402. Fax: +81-86-235-7404. E-mail: eijimatu@md.okayama-u.ac.jp

ISSN 1740-2522 print/ISSN 1740-2530 online © 2003 Taylor & Francis Ltd
DOI: 10.1080/10446670310001642113

several elements in the protein C, protein S anticoagulant system (Merrill *et al.*, 1999). $\beta_2$GPI's fifth domain contains a patch of positively charged amino acids that likely represents the binding region for phospholipids (Bouma *et al.*, 1999; Hoshino *et al.*, 2000).

Venous thromboembolic complications represent the most common clinical finding in APS patients (Harris *et al.*, 1986; Ginsburg *et al.*, 1992; Bick and Baker, 1999). However, over 25% of the patients enrolled into a European cohort of 1000 APS patients presented an arterial thrombotic event (myocardial infarction, cerebrovascular accident, angina, etc.) as the initial clinical manifestation (Cervera *et al.*, 2002). More recently, the premature (or accelerated) development of atherosclerosis has been recognized in autoimmune patients (Ward, 1999; Aranow and Ginzler, 2000; van Doornum *et al.*, 2002). The traditional risk factors for atherosclerosis failed to account for these changes (Esdaile *et al.*, 2001). Increased levels of autoantibodies against oxidized low-density lipoprotein (oxLDL), phospholipids, and Lp(a), have been proposed as alternative mechanisms as well as certain biochemical and genetic abnormalities (Lockshin *et al.*, 2001). Oxidation of LDL (oxLDL) plays an important pathogenic role in early events leading to atherosclerosis (Berliner and Heinecke, 1996; Steinberg, 1997). oxLDL is a pro-inflammatory chemotactic agent for macrophages and T lymphocytes, which have a central role in atherogenesis (McMurray *et al.*, 1993). In addition, oxLDL has been found in human and rabbit atherosclerotic lesions (Yla-Herttuala *et al.*, 1989), and shown to be an immunogen producing autoantibodies in patients with autoimmune disorders, such as systemic lupus erythematosus (SLE) and APS (Salonen *et al.*, 1992; Vaarala *et al.*, 1993). The participation of the immune system in the development of atherosclerosis is becoming apparent and some antiphospholipid antibodies may also be possible participants (Vaarala, 1996; Romero *et al.*, 1998; Tinahones *et al.*, 1998).

$\beta_2$GPI has also been localized in human atherosclerotic lesions by immunohistochemical staining (George *et al.*, 1999), which suggests a role of $\beta_2$GPI (and antiphospholipid antibodies, i.e. anti-$\beta_2$GPI antibodies) in atherosclerosis. In 1997, we (Hasunuma *et al.*, 1997) reported that $Cu^{2+}$-oxLDL, unlike native LDL, binds to $\beta_2$GPI. *In vitro* macrophage uptake of oxLDL was slightly decreased in the presence of $\beta_2$GPI, as compared to oxLDL alone. In contrast, the addition of an antiphospholipid antibody, i.e. $\beta_2$GPI-dependent aCL (or anti-$\beta_2$GPI), together with $\beta_2$GPI, resulted in a significant increase of oxLDL uptake by macrophages. It is well-known that oxLDL uptake by macrophages is inhibited with polyinosinic acid, a scavenger receptor blocker. However, the increased $\beta_2$GPI and anti-$\beta_2$GPI antibody dependent uptake was not affected by polyinosinic acid and it is most possible that macrophage Fc$\gamma$ receptors were involved in the binding. This mechanism may be relevant to the development of atherosclerosis in patients with APS. The $\beta_2$GPI-specific ligand on the oxLDL particles (oxLig-1, 7-ketocholesteryl-9-carboxynonanoate) responsible for the oxLDL interaction with $\beta_2$GPI has been isolated and identified. Increased macrophage uptake of liposomes (as a model of oxLDL) has also been reported when oxLig-1/$\beta_2$GPI/antibody complexes were applied (Kobayashi *et al.*, 2001; Liu *et al.*, 2002). Most recently, we have reported that oxidatively modified LDL interacts *in vivo* with $\beta_2$GPI, and detected $\beta_2$GPI/oxLDL complexes, autoantibodies against $\beta_2$GPI/oxLig-1 complexes, and IgG immune complexes containing $\beta_2$GPI and oxLDL in serum samples from SLE and APS patients (Kobayahsi *et al.*, 2003).

In the present study, serum levels of $\beta_2$GPI/oxLDL complexes and IgG anti-$\beta_2$GPI/oxLig-1 autoantibodies were measured in patients with APS, and their association with clinical manifestations of APS was assessed. Our results indicate that oxidation of LDL leads the complex formation with $\beta_2$GPI, and that these complexes commonly appear in the blood stream of patients with APS as well as in SLE patients with or without APS. However, autoantibodies against $\beta_2$GPI/oxLig-1 were only generated in APS patients. Further, these antibodies showed a stronger correlation with arterial thrombosis when compared to venous thrombosis. These results may indicate etiological importance of IgG anti-$\beta_2$GPI/oxLDL (oxLig-1) autoantibodies in the development of atherosclerosis in APS patients.

### MATERIALS AND METHODS

### Patients

Serum samples from 150 patients were utilized in the study. One hundred samples were obtained from APS patients enrolled in the Registry for the APS (Oklahoma Medical Research Foundation, Oklahoma City, OK—www.slrapls.org). The clinical diagnosis of APS was based on the Sapporo criteria for the classification of APS (Wilson *et al.*, 1999). All patients had a positive lupus anticoagulant and/or IgG $\beta_2$GPI-dependent aCL ELISA result on 2 or more occasions. Twenty-four patients were classified as primary APS and 76 as secondary APS to SLE. Eighty-eight of the APS patients were females and 12 males. The mean age was 44.6 years (range 18–82 years). A separate population of 50 patients meeting the 1982 ACR criteria for SLE (Tan *et al.*, 1982), with no history of antiphospholipid antibodies, was used as control. In addition, 43 serum samples from healthy blood bank donors were also included in this study as controls.

Three major clinical manifestations for APS were recorded: venous thrombosis, arterial thrombosis and pregnancy morbidity. Venous thrombotic events included deep-vein thrombosis (DVT), pulmonary embolism (PE) and superficial phlebitis confirmed by Doppler ultrasound, venography or ventilation–perfusion scanning. Arterial thrombotic events included myocardial infarction (MI), cerebrovascular accident (CVA) or peripheral arterial thrombosis. Pregnancy morbidity was evaluated separately,

TABLE I Patients' clinical characteristics

| *Patients* | *n* |
|---|---|
| Primary APS | 24 |
| Secondary APS (to SLE) | 76 |
| SLE without APS (controls) | 50 |
| Total | 150 |
| *APS classification* | |
| Total thrombosis | 85 |
| Arterial thrombosis | 45 |
| Arterial thrombosis only | 31 |
| Arterial + venous thrombosis | 14 |
| Venous thrombosis only | 40 |
| Pregnancy morbidity only | 15 |

including pregnancy loss after 10 weeks of gestation and/or late pregnancy complications as previously defined (Wilson *et al.*, 1999). Fourteen of the APS patients had a history of thrombocytopenia (platelet count $< 100{,}000\,mm^3$). In all cases, thrombocytopenia was present in combination with at least one of the above clinical manifestations, since the Sapporo criteria were used. The clinical characteristics and classification of the APS patients studied are summarized in Table I.

The Registry for the APS has been approved and monitored by the Internal Review Boards (IRB) of the Oklahoma Medical Research Foundation, New York University Medical Center and (previously) Saint Luke's-Roosevelt Hospital Center in New York City. Informed consent was given to all participants according to FDA/ICH guidelines and institutional requirements. The current project was pre-approved by the Registry Advisory Board. A material transfer agreement and inter-institutional assurances were initiated in accordance with current regulations.

### Monoclonal Antibodies

The following monoclonal antibodies were used to develop and calibrate the ELISA tests for measuring $\beta_2$GPI/oxLDL complex and anti-$\beta_2$GPI/oxLig-1 antibodies: WB-CAL-1 monoclonal antibody reactive to $\beta_2$GPI (IgG2a, $\kappa$) derived from a NZW x BXSB F1 mouse, a spontaneous model of APS (Hashimoto *et al.*, 1992), and EY2C9 monoclonal anti-$\beta_2$GPI antibody (IgM) established from peripheral blood lymphocytes of APS patients (Ichikawa *et al.*, 1994). Both monoclonal antibodies bind only to $\beta_2$GPI complexed with $Cu^{2+}$-oxLDL and negatively-charged phospholipid, such as CL and phosphatidylserine, but not to monomeric (free) $\beta_2$GPI in solution. 1D2 (Yamasa Corporation, Choshi, Japan) is an IgG murine monoclonal antibody specific for human ApoB-100 and the antibody binding is not affected by the oxidation of LDL.

### Purification of Human $\beta_2$GPI

Human $\beta_2$GPI was purified from fresh normal plasma as previously described (Finlayson and Mushinski, 1967) with slight modifications. Briefly, human plasma was first precipitated with 70% perchloric acid, extensively dialyzed against Tris/NaCl buffer (pH 8.0) and concentrated before loading into a heparin column (Amersham Biosciences, Piscataway, NJ). Pooled $\beta_2$GPI fractions were again dialyzed against sodium acetate/NaCl buffer (pH 4.8) and concentrated. This preparation was then loaded into a CM cellulose column (Sigma-Aldrich, St. Louis, MO) and $\beta_2$GPI fractions were pooled, dialyzed against sodium acetate/NaCl buffer, concentrated at approximately 1 mg/ml and stored at $-70°C$ until use. The reactivity of $\beta_2$GPI was checked by ELISA and the purity was assessed by SDS-PAGE.

### LDL Purification and Oxidation

LDL was isolated by ultracentrifugation of fresh normal human plasma in EDTA/KBr solutions as described (Havel *et al.*, 1955). LDL ($d = 1.019-1.063$ g/ml) was adjusted to a concentration of 100 μg/ml based on protein concentration. The LDL fraction was oxidized with 5 μM $CuSO_4$ in 10 mM phosphate buffer containing, 150 mM NaCl, pH 7.4 (PBS) at 37°C for 12 h. Oxidation was terminated by the addition of EDTA (at a final concentration of 1 mM), and extensively PBS containing EDTA. The degree of oxidation was measured using the thiobarbituric acid reactive substance (TBARS) procedure (Ohkawa *et al.*, 1979).

### ELISA Procedure for $\beta_2$GPI/oxLDL Complexes

In the present study, the ELISA for $\beta_2$GPI/oxLDL complexes was performed in the presence of $\beta_2$GPI to ensure the detection of all possible forms of oxLDL. oxLDL is predominantly present as a complex with $\beta_2$GPI but it may be present as free oxLDL. Monoclonal antibody against complexed $\beta_2$GPI (WB-CAL-1) was coated onto 96-well microtiter plate (Immunlon 2HB, Dynex Technologies Inc., Chantilly, VA) by incubating 50 μl/well of 5 μg/ml of WB-CAL-1 in PBS, pH 7.4, overnight at 2–4°C. The plate was blocked with PBS containing 1% non-fat dry milk (nfdm) for 1 h. Fifty microliters of 30 μg/ml of human $\beta_2$GPI in PBS was added to each well, followed by 50 μl of the serum samples diluted at 1:25 in PBS-nfdm, and incubated for 2 h at room temperature. The wells were washed 4 times with PBS containing 0.05% Tween-20 between each step. Biotinylated 1D2 (anti-human ApoB-100) antibody diluted in PBS-nfdm was added to the wells and incubated for 1 h at room temperature, followed by horseradish peroxidase (HRP)-streptavidin. Color was developed with tetramethylbenzidine (TMB)/$H_2O_2$ and the reaction was stopped with 0.36N sulfuric acid. Optical density was measured at 450 nm. Serum oxLDL concentration (indicated as U/ml) was calculated as a complex with $\beta_2$GPI, against a reference curve built with 2-fold serial dilutions of a known concentration of oxLDL added to wells containing $\beta_2$GPI. The unit value was arbitrarily derived from the concentration of the material used in the reference curve.

A normal cut-off value for the assay was established at 23 U/ml by testing 43 samples from healthy blood donors (mean + 3 standard deviations).

### ELISA for IgG Anti-$\beta_2$GPI/oxLig-1 Antibodies

The ELISA procedure used in the study has been previously described by Kobayashi *et al.* (2001) with slight modification. Fifty microliters of 100 μg/ml of oxLig-1 (7-ketocholesteryl-9-carboxynonanoate) in ethanol was coated onto Immunlon 2HB plates by evaporation. The synthesis and characterization of oxLig-1 has been recently reported (Kobayashi *et al.*, 2001; Liu *et al.*, 2002). The plate was blocked with 1% BSA for 1 h at room temperature and washed. Fifty microliters of 30 μg/ml of human $\beta_2$GPI in PBS containing 0.3% BSA was added to the oxLig-1 coated wells to allow complex formation. Fifty microliters of serum or plasma samples diluted 1:100 in PBS containing 0.3% BSA were subsequently added to the wells and incubated for 1 h at room temperature. The wells were washed 4 times with PBS containing 0.05% Tween-20 between steps. Diluted HRP-conjugated anti-human IgG antibody was added to the wells and incubated for 1 h. Color was developed with TMB/$H_2O_2$ and the reaction stopped with 0.36 N sulfuric acid. Optical density was measured at 450 nm. To establish the initial performance of the assay and to select a strong reactive sample to be used as control, monoclonal antibody, EY2C9, and HRP-conjugated anti-human IgM antibody were used. Level of IgG anti-$\beta_2$GPI/oxLig-1 antibodies in samples (expressed in U/ml as defined above) was calculated against the curve prepared with a selected serum positive sample. A normal cut-off value for the assay was established at 10 U/ml by testing 43 samples from healthy blood donors (mean + 3 standard deviations).

### ELISA for aCL and Anti-$\beta_2$GPI Antibodies

All APS samples were tested for IgG aCL and anti-$\beta_2$GPI antibodies on commercially available ELISA test kits (Corgenix Inc., Westminster, CO), following the manufacturer's instructions. The aCL ELISA test uses exogenous bovine $\beta_2$GPI thus measuring $\beta_2$GPI-dependent antibodies. The anti-$\beta_2$GPI ELISA test uses purified human $\beta_2$GPI as antigen in the absence of exogenous phospholipids.

### Statistical Analysis

Statistical analysis was performed with a SigmaStat program (SPSS Science Inc., Chicago, IL). Student's *t* test was performed to compare the results between different groups and Chi-square test was used to assess the relationship between antibodies and clinical manifestations. Sensitivity, specificity, positive predictive value (PPV) and odds ratio of anti-$\beta_2$GPI/oxLig-1 antibodies were calculated by 2 × 2 contingency table analysis. Ninety-five percent confidence intervals for odds ratios were also calculated. Pearson's product moment correlation was performed to assess the association of individual values between variables. A $p$ value of 0.05 or less was considered as significant.

### RESULTS

### Serum Levels of $\beta_2$GPI/oxLDL Complexes

Figure 1 shows that most APS patients had elevated serum levels of $\beta_2$GPI/oxLDL complexes with a mean level of 96.7 ± 72.3 U/ml, while none of the healthy controls reacted above the cut-off (mean 12.4 ± 3.7 U/ml, $p = 5.8 \times 10^{-9}$). The mean complex level of 24 primary APS patients was 105.3 ± 84.1 U/ml, similar to the mean of 76 patients with secondary APS to SLE (93.9 ± 68.5 U/ml) and the mean level of 50 SLE patients without APS (88.5 ± 76.1 U/ml). The mean complex level for each APS subgroup was not statistically different: 98.9 ± 75.4 U/ml for arterial thrombosis ($n = 45$), 91.3 ± 57.7 U/ml for venous one ($n = 40$) and 104.2 ± 98.3 U/ml for pregnancy morbidity ($n = 15$). However, the mean complex level of 31 patients with arterial thrombosis only was 83.6 ± 64.3 U/ml, significantly lower ($p = 0.039$), as compared with the mean level of 14 patients with both arterial and venous thrombosis (132.8 ± 88.9 U/ml). These results indicate that oxidation of LDL leads the complex formation with $\beta_2$GPI and the complexes commonly appear in APS patients and SLE patients with/or without APS. In addition, $\beta_2$GPI/oxLDL complexes were particularly high in a subgroup with apparent increased vasculopathy as evidence by both arterial and venous thrombotic history.

### Serum IgG Anti-$\beta_2$GPI/oxLig-1 Antibodies

Thirty-six percent of the APS patients had elevated levels of IgG anti-$\beta_2$GPI/oxLig-1 antibodies with a mean level of 22.5 ± 64.9 U/ml, significantly higher as compared with SLE patients without APS (9.1 ± 5.1 U/ml, $p = 0.02$) and to healthy controls (5.7 ± 1.4 U/ml, $p = 0.005$). There was no difference between primary and secondary APS with regard to the antibody levels. The mean IgG anti-$\beta_2$GPI/oxLig-1 level of each subgroup was: 23.4 ± 41.9 U/ml for arterial thrombosis ($n = 45$) with 40% classified as positive, 12.3 ± 16.5 U/ml for venous ($n = 39$) with 36% positives, and 8.6 ± 6.3 U/ml for pregnancy morbidity ($n = 15$) with 20% positives (Fig. 2). The mean level of the venous thrombosis ($p = 0.05$) and the pregnancy morbidity ($p = 0.01$) subgroups were statistically lower as compared with that of arterial thrombosis subgroup. These results indicate significantly higher serum levels of IgG anti-$\beta_2$GPI/oxLig-1 antibodies in primary and secondary APS patients as compared with SLE patients without APS and healthy controls. In addition, APS patients with a history of arterial thrombosis had significantly higher antibody levels,

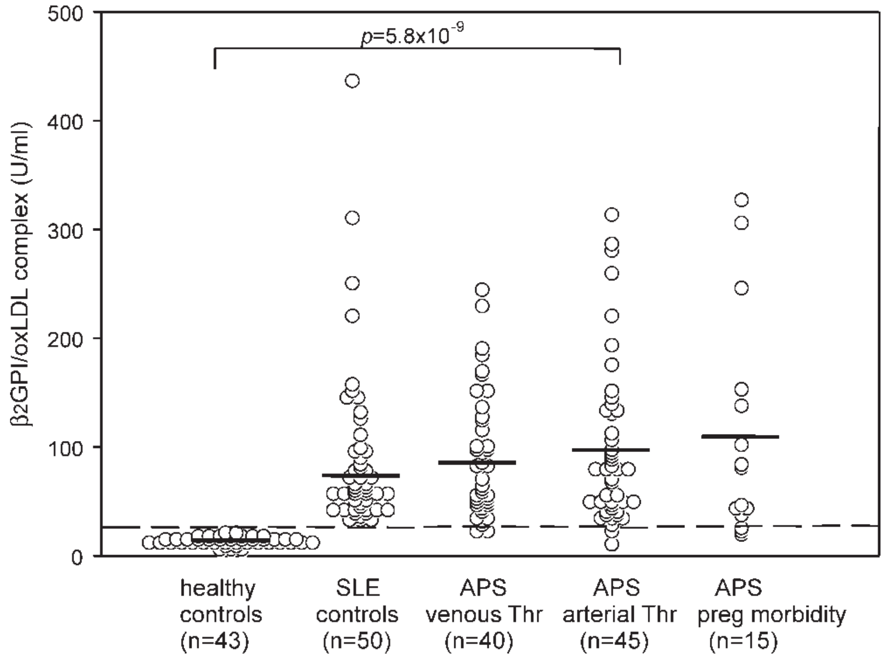

FIGURE 1 Serum levels of $\beta_2$GPI/oxLDL complexes measured by ELISA in healthy controls, SLE without clinical or serologic manifestations of APS (diseased controls), and 100 APS patients classified into groups according to their history of arterial thrombosis, venous thrombosis or pregnancy morbidity. The cut-off (horizontal broken line) was established at 23 U/ml (mean + 3 standard deviations from 43 healthy subjects). The horizontal solid lines indicate the mean $\beta_2$GPI/oxLDL level of each group.

as compared with patients with venous thrombosis or pregnancy morbidity.

### Relationship of IgG Anti-$\beta_2$GPI/oxLig-1 Antibodies with aCL and Anti-$\beta_2$GPI Antibodies

Due to the prominent presence of $\beta_2$GPI in the antigenic mixture used to detect IgG anti-$\beta_2$GPI/oxLig-1 antibodies, the relationship of these antibodies with $\beta_2$GPI-dependent antiphospholipid antibodies was evaluated. Figure 3 basically shows a good correlation of IgG anti-$\beta_2$GPI/oxLig-1 antibodies with (A) IgG aCL, and (B) with anti-$\beta_2$GPI antibodies in 100 APS patients ((A) $r = 0.832$, $p < 0.001$ and (B) $r = 0.688$, $p < 0.001$, respectively). However, The graph on the relationship of

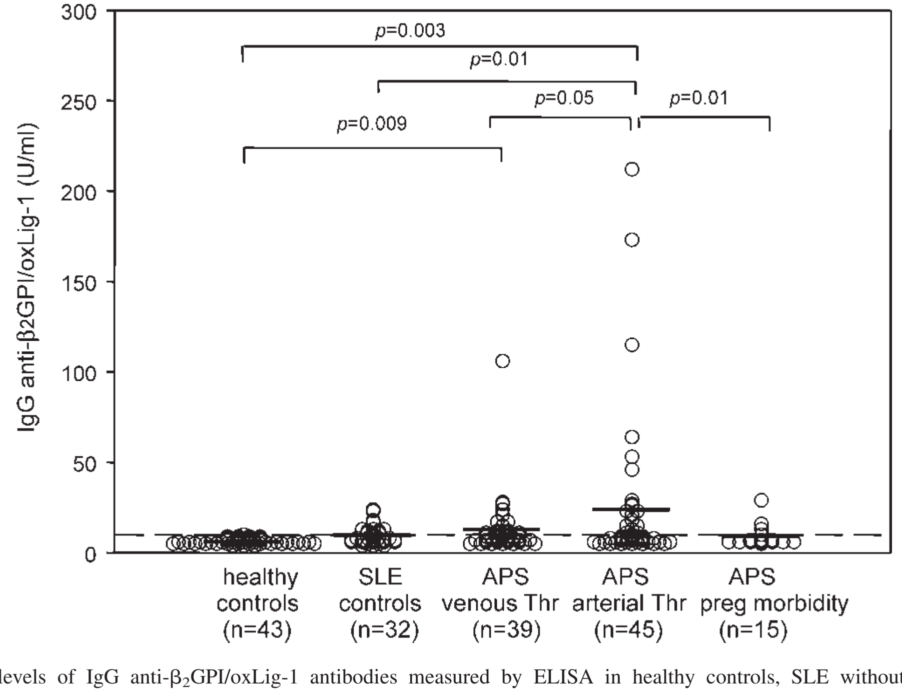

FIGURE 2 Serum levels of IgG anti-$\beta_2$GPI/oxLig-1 antibodies measured by ELISA in healthy controls, SLE without clinical or serologic manifestations of APS (diseased controls), and 100 APS patients classified into groups according to their history of arterial thrombosis, venous thrombosis or pregnancy morbidity. The cut-off (horizontal broken line) was established at 10 U/ml (mean + 3 standard deviations from 43 healthy subjects). The horizontal solid lines indicate the mean IgG anti-$\beta_2$GPI /oxLig-1 antibody level of each group.

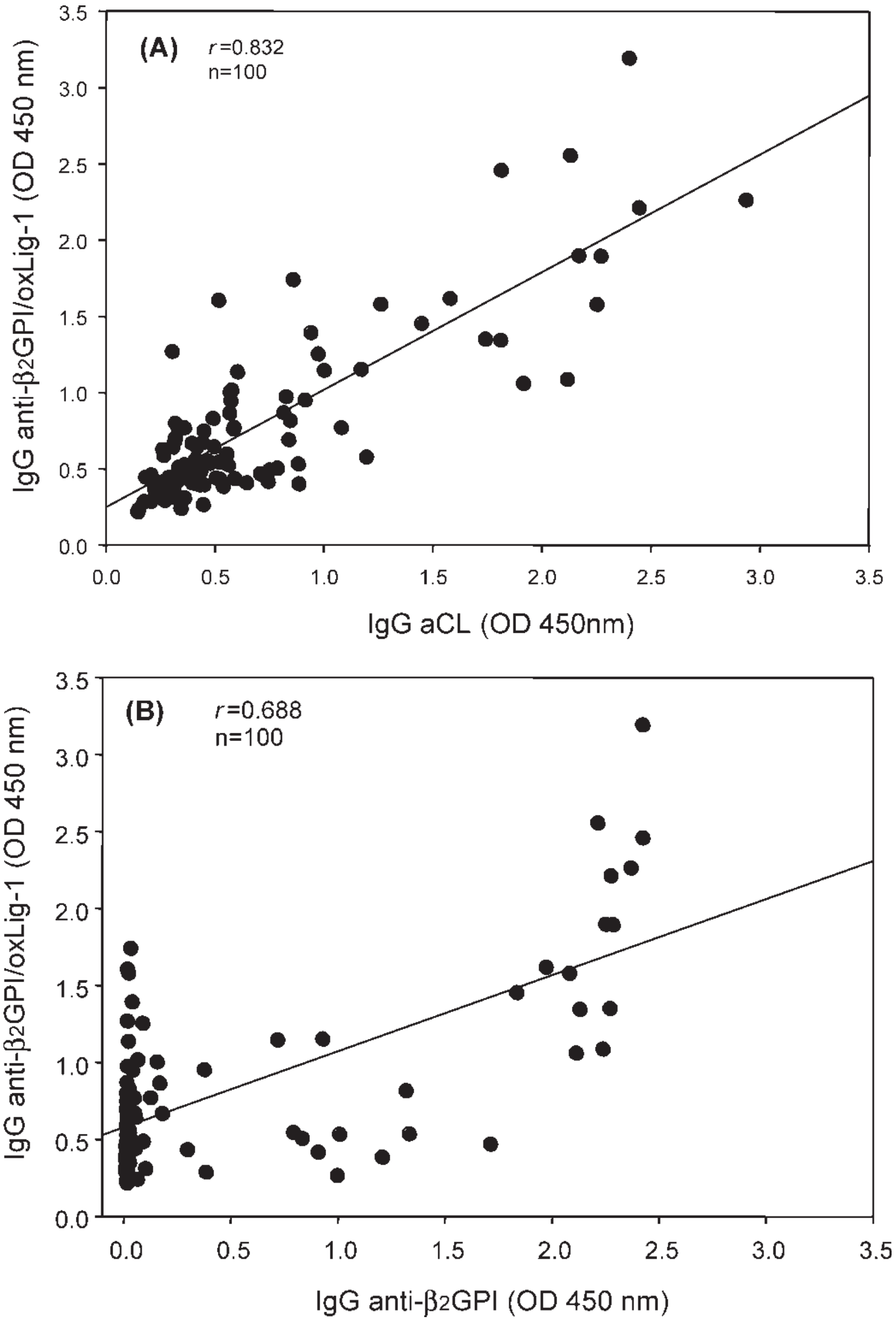

FIGURE 3 Correlation between IgG anti-$\beta_2$GPI/oxLig-1 antibodies and antiphospholipid antibodies determined by ELISA in 100 APS patients. (A) IgG anti-$\beta_2$GPI/oxLig-1 antibodies versus IgG anticardiolipin antibodies (aCL); (B) IgG anti-$\beta_2$GPI/oxLig-1 antibodies versus IgG anti-$\beta_2$GPI antibodies. The straight line represents the best-fit linear regression.

IgG anti-$\beta_2$GPI/oxLig-1 versus anti-$\beta_2$GPI antibodies also showed a little dislocating distribution pattern. This pattern may suggest the presence of distinct populations of antibodies, some are much reactive for $\beta_2$GPI directly and others are to $\beta_2$GPI/oxLig-1. Twelve (27%) of the APS patients in the arterial thrombosis subgroup had antibodies reacting to both $\beta_2$GPI and $\beta_2$GPI/oxLig-1, while only 4 (10%) in the venous thrombosis and none in the pregnancy morbidity groups had this dual reactivity.

In comparing the arterial, venous and pregnancy morbidity subgroups, the correlation between IgG anti-$\beta_2$GPI/oxLig-1 antibodies with IgG aCL, and between IgG anti-$\beta_2$ GPI/oxLig-1 anitbodies and IgG anti-$\beta_2$GPI antibodies was strongest in the arterial thrombosis ($r = 0.807$ and $r = 0.629$ respectively), as compared with the venous thrombosis ($r = 0.760$ and $r = 0.559$) and the pregnancy morbidity subgroups ($r = 0.038$ and $r = 0.134$). Thus, IgG anti-$\beta_2$GPI/oxLig-1 antibodies

TABLE II Association between IgG anti-$\beta_2$GPI/oxLig-1 antibodies and thrombosis or pregnancy morbidity in APS patients

| APS manifestation (*n*) | Sensitivity (%) | PPV (%) | Chi-square (*p*) | OR (95% CI) |
|---|---|---|---|---|
| Total thrombosis (85) | 38.8 | 94.3 | 0.001 | 9.5 (2.1–42.5) |
| Arterial thrombosis (45) | 40.0 | 90.0 | 0.002 | 10 (2.1–47.1) |
| Venous thrombosis (40) | 37.5 | 88.2 | 0.005 | 9 (1.9–43.1) |
| Pregnancy morbidity (15) | 20.0 | 60.0 | 0.309* | 3.7 (0.5–25.3) |

PPV, positive predictive value; OR, odds ratio; CI, 95% confidence interval.
*not statistically significant.

may represent a distinct subset of antiphospholipid antibodies that are particularly associated with arterial thrombosis.

### Comparative Clinical Performance

The clinical performance (relative sensitivity and positive predictive value—PPV) of IgG anti-$\beta_2$GPI/oxLig-1 antibodies for the history of thrombosis (arterial and venous) and pregnancy morbidity in APS patients was evaluated by $2 \times 2$ contingency table analysis. Table II shows that IgG anti-$\beta_2$GPI/oxLig-1 antibodies were 38.6% sensitive for total thrombosis (arterial and venous combined) with a PPV of 94% ($p = 0.001$). The specificity of this antibody for total thrombosis was 93.7%. The PPV for arterial thrombosis was 90% and for venous thrombosis 88% ($p = 0.002$ and 0.005, respectively). The relative sensitivity for pregnancy morbidity was 20% with a PPV of 60% ($p = 0.309$). These results indicate that IgG anti-$\beta_2$GPI/oxLig-1 antibodies are found predominantly in those autoimmune patients who have a history of vasculopathy, with a stronger association for arterial than venous thrombosis in patients with APS.

### DISCUSSION

The cholesterol that accumulates in macrophage-derived foam cells of atherosclerotic lesions is derived from circulating lipoproteins, mainly LDL, but LDL must be modified before it can induce foam cell formation (Ross, 1999). Oxidation of LDL is an effective mechanism that modifies LDL, increasing its macrophage uptake via scavenger receptors and intracellular accumulation. Several studies have demonstrated that atherosclerosis is an inflammatory disease, involving the dysregulation of cholesterol homeostasis by aberrant interactions between lipid-modulating elements and mediators of inflammation (Steinberg, 2002). Although the initiating inflammatory factor(s) remain unknown, likely candidates include oxLDL, immunological injury, homocysteine and infectious agents. An active role of antibodies in this process has been proposed (Virella *et al.*, 2002) as recent prospective studies have indicated that $\beta_2$GPI-dependent aCL or anti-$\beta_2$GPI antibodies are associated with MI and stroke in men (Vaarala, 1998; Brey *et al.*, 2001).

Our results indicate that oxidation of LDL is a common occurrence in APS and SLE patients without APS, and has demonstrated the presence of circulating $\beta_2$GPI/oxLDL complexes in these patients (Fig. 1). Although it can be hypothesized that this might be related to chronic inflammation of the vasculature that occurs in autoimmune patients, the mechanism(s) for the increased oxidation of LDL found here are not known. $\beta_2$GPI binds to oxLDL, not to native LDL, possibly promoting its clearance from circulation (Hasunuma *et al.*, 1997) and preventing thrombus formation. Circulating $\beta_2$GPI/oxLDL complexes have been implicated as atherogenic autoantigens, and their presence may represent a risk factor or an indirect but significant contributor for thrombosis and atherosclerosis (Kobayahsi *et al.*, 2003) in an autoimmune background. As numerous interacting inflammatory, oxidative and coagulation factors are thought to contribute to the development of atherosclerosis, the oxidative modification of LDL may play a role in the initiation, progression and terminal events in these vascular lesions (Ross, 1999).

The high-density lipoprotein (HDL)-associated enzyme paraoxonase (PON) has anti-oxidant activity that protects LDL from oxidation (Durrington *et al.*, 2001). Decreased PON activity has been reported in patients with high levels of aCL (Lambert *et al.*, 2000). Furthermore, IgG anti-$\beta_2$GPI antibodies have been associated with reduced PON activity in SLE and primary APS patients (Delgado-Alves *et al.*, 2002). PON activity is also known to increase with lipid-lowering drugs (Belogh *et al.*, 2001; Senti *et al.*, 2001), and in one study, cholesterol-lowering statins prevented the *in vitro* endothelial cell activation normally induced by anti-$\beta_2$GPI antibodies (Meroni *et al.*, 2001). Antioxidant treatment for 4–6 weeks has been observed to decrease the titer of circulating aCL antibodies in SLE and APS patients (Ferro *et al.*, 2002). Vascular injury as seen in autoimmune patients may affect PON activity or any other anti-oxidant mechanism, triggering LDL oxidative changes. Taken together, these findings provide additional support to the hypothesis that oxidative stress plays an important role in antiphospholipid antibody production and development of thrombosis in APS.

The mean level of IgG anti-$\beta_2$GPI/oxLig-1 antibodies was highest in APS patients with arterial thrombosis (Fig. 2). The coexistence of these autoantibodies with $\beta_2$GPI/oxLDL complexes, suggest that these two elements interact perhaps forming circulating immune complexes. This observation along with the increased macrophage uptake of $\beta_2$GPI/oxLDL complexes in the presence of anti-$\beta_2$GPI/oxLig-1 antibodies, provides a possible explanation for the accelerated development of atherosclerosis in autoimmune patients. Two groups (Zhao *et al.*, 2001; Kobayahsi *et al.*, 2003) using similar assay systems have recently shown increased serum levels of oxLDL and antibodies to oxLDL in APS patients with history of arterial thrombotic events. It is possible that APS patients also present immune complexes ($\beta_2$GPI/oxLDL/antibody). The ELISA system used in this study seems to detect only free (unbound) antibodies to $\beta_2$GPI/oxLDL (oxLig-1) complexes. Although preliminary, our results suggest that IgG anti-$\beta_2$GPI/oxLDL (oxLig-1) antibodies may represent a distinct subset of antiphospholipid antibodies and that they may coexist with other antibodies. IgG anti-$\beta_2$GPI/oxLDL (oxLig-1) antibodies appear to be a useful serologic marker with high specificity for APS and might possibly have a pathogenic role in atherosclerotic risk in autoimmune patients.

### *References*

Aranow, C. and Ginzler, E.M. (2000) "Epidemiology of cardiovascular disease in systemic lupus erythematosus", *Lupus* **9**, 166–169.

Belogh, Z., Seres, I., Harangi, M., Kovacs, P., Kakuk, G. and Paragh, G. (2001) "Gemfibrozil increases paraoxonase activity in type 2 diabetic patients: a new hypothesis of the beneficial action of fibrates?", *Diabetes Metab.* **27**, 604–610.

Berliner, J.A. and Heinecke, J.W. (1996) "The role of oxidized lipoproteins in atherogenesis", *Free Radic. Biol. Med.* **20**, 707–727.

Bick, R.L. and Baker, W.F. (1999) "Antiphospholipid syndrome and thrombosis", *Semin. Thromb. Hemost.* **25**, 333–350.

Bouma, B., de Groot, P.G., van den Elsen, J.M.H., *et al.* (1999) "Adhesion mechanism of human $\beta_2$-glycoprotein I to phospholipids based on its crystal structure", *EMBO J.* **18**, 5166–5174.

Brey, R.L., Abbott, R.D., Curb, J.D., *et al.* (2001) "$\beta_2$-glycoprotein I dependent anticardiolipin antibodies and the risk of ischemic stroke and myocardial infarction", *Stroke* **32**, 1701–1706.

Cervera, R., Piette, J.C., Font, J., *et al.* (2002) "Antiphospholipid syndrome. Clinical and immunologic manifestations and patterns of disease expression in a cohort of 1,000 patients", *Arthritis Rheum.* **46**, 1019–1027.

Delgado-Alves, J., Ames, P.R.J., Donohue, S., *et al.* (2002) "Antibodies to high-density lipoprotein and $\beta_2$-glycoprotein I are inversely correlated with Paraoxonase activity in Systemic lupus erythematosus and primary antiphospholipid syndrome", *Arthritis Rheum.* **46**, 2686–2694.

Durrington, P.N., Mackness, B. and Mackness, M.I. (2001) "Paraoxonase and atherosclerosis", *Arterioscler. Thromb. Vasc. Biol.* **21**, 473–480.

Esdaile, J.M., Abrahamowicz, M., Grodzicky, T., *et al.* (2001) "Traditional Framingham risk factors fail to fully account for accelerated atherosclerosis in systemic lupus erythematosus", *Arthritis Rheum.* **44**, 2331–2337.

Ferro, D., Iuliano, L., Violi, F., Valesini, G. and Conti, F. (2002) "Antioxidant treatment decreases the titer of circulating anticardiolipin antibodies", *Arthritis Rheum.* **46**, 3110–3112.

Finlayson, J.S. and Mushinski, J.F. (1967) "Separation of subfractions of human $\beta_2$-glycoprotein I", *Biochim. Biophys. Acta* **147**, 413–420.

George, J., Harats, D., Gilburd, B., *et al.* (1999) "Immunolocalization of $\beta_2$-glycoprotein I (apolipoprotein H) to human atherosclerotic plaques: potential implications for lesion progression", *Circulation* **99**, 2227–2230.

Gharavi, A.E., Harris, E.N., Asherson, R.A. and Hughes, G.R.V. (1987) "Anticardiolipin antibodies-isotype distribution and phospholipid specificity", *Ann. Rheum. Dis.* **46**, 1–6.

Ginsburg, K.S., Liang, M.H., Newcomer, L., *et al.* (1992) "Anticardiolipin antibodies and the risk for ischemic stroke and venous thrombosis", *Ann. Intern. Med.* **117**, 997–1002.

Harris, E.N., Chan, J.K.H., Asherson, R.A. and Hughes, G.R.V. (1986) "Thrombosis, recurrent fetal loss and thrombocytopenia-predictive value of the anticardiolipin antibody test", *Arch. Intern. Med.* **146**, 2153–2156.

Hashimoto, Y., Kawamura, M., Ichikawa, K., *et al.* (1992) "Anticardiolipin antibodies in NZW × BXSB F1 mice: a model of antiphospholipid syndrome", *J. Immunol.* **149**, 1063–1068.

Hasunuma, Y., Matsuura, E., Makita, Z., Katahira, T., Nishi, S. and Koike, T. (1997) "Involvement of $\beta_2$-glycoprotein I and anticardiolipin antibodies in oxidatively modified low density lipoprotein uptake by macrophages", *Clin. Exp. Immunol.* **107**, 569–573.

Havel, R.J., Eder, H.A. and Bragdon, J.H. (1955) "The distribution and chemical composition of ultracentrifugally separated lipoproteins in human serum", *J. Clin. Investig.* **43**, 1345–1353.

Hoshino, M., Hagihara, Y., Nishii, I., Yamazaki, T., Kato, H. and Goto, Y. (2000) "Identification of the phospholipid-binding site of human $\beta_2$-glycoprotein I domain V by heteronuclear magnetic resonance", *J. Mol. Biol.* **304**, 927–939.

Hughes, G.R.V., Harris, E.N. and Gharavi, A.E. (1986) "The anticardiolipin syndrome", *J. Rheumatol.* **13**, 486–489.

Ichikawa, K., Khamashta, M.A., Koike, T., Matsuura, E. and Hughes, G.R.V. (1994) "$\beta_2$-glycoprotein I reactivity of monoclonal anticardiolipin antibodies from patients with the antiphospholipid syndrome", *Arthritis Rheum.* **37**, 1453–1461.

Kobayashi, K., Matsuura, E., Liu, Q., *et al.* (2001) "A specific ligand for $\beta_2$-glycoprotein I mediates autoantibody-dependent uptake of oxidized low density lipoprotein by macrophages", *J. Lipid Res.* **42**, 697–709.

Kobayahsi, K., Kishi, M., Atsumi, T., *et al.* (2003) "Circulating oxidized low density lipoprotein forms complexes with $\beta_2$-glycoprotein I: implication as an atherogenic autoantigen", *J. Lipid Res.* **44**, 716–726.

Lambert, M., Boullier, A., Hachulla, E., *et al.* (2000) "Paraoxonase activity is dramatically decreased in patients positive for anticardiolipin antibodies", *Lupus* **9**, 299–300.

Liu, Q., Kobayashi, K., Furukawa, J., *et al.* (2002) "ω-Carboxyl variants of 7-ketocholesteryl esters are ligands for $\beta_2$-glycoprotein I and mediate antibody-dependent uptake of oxidized LDL by macrophages", *J. Lipid Res.* **43**, 1486–1495.

Lockshin, M.D., Salmon, J.E. and Roman, M.J. (2001) "Atherosclerosis and lupus: a work in progress", *Arthritis Rheum.* **44**, 2215–2217.

Matsuura, E., Igarashi, Y., Fujimoto, M., Ichikawa, K. and Koike, T. (1990) "Anticardiolipin cofactor(s) and differential diagnosis of autoimmune diseases", *Lancet* **336**, 177–178.

Matsuura, E., Igarashi, Y., Yasuda, T., Triplett, D.A. and Koike, T. (1994) "Anticardiolipin antibodies recognize $\beta_2$-glycoprotein I structure altered by interacting with an oxygen modified solid phase surface", *J. Exp. Med.* **179**, 457–462.

McMurray, H.F., Parthasarathy, S. and Steinberg, D. (1993) "Oxidatively modified low density lipoprotein is a chemoattractant for human T lymphocytes", *J. Clin. Investig.* **92**, 1004–1008.

McNeil, H.P., Simpson, R.J., Chesterman, C.N. and Krilis, S.A. (1990) "Antiphospholipid antibodies are directed against a complex antigen that includes a lipid-binding inhibitor of coagulation: $\beta_2$-glycoprotein I (apolipiprotein H)", *Proc. Natl Acad. Sci. USA* **87**, 4120–4124.

Meroni, P.L., Raschi, E., Testoni, C., *et al.* (2001) "Statins prevent endothelial cell activation induced by antiphospholipid (anti-$\beta_2$-glycoprotein I) antibodies. Effect on the proadhesive and proinflammatory phenotype", *Arthritis Rheum.* **44**, 2870–2878.

Merrill, J.T., Zhang, H.W., Shen, C., *et al.* (1999) "Enhancement of Protein S anticoagulant function by $\beta_2$-glycoprotein I, a major target antigen of antiphospholipid antibodies: $\beta_2$-glycoprotein I interferes with binding of Protein S to its plasma inhibitor, C4b-binding protein", *Thromb. Haemost.* **81**, 748–757.

Ohkawa, H., Ohishi, N. and Yagi, K. (1979) "Assay for lipid peroxides in animal tissues by thiobarbituric acid reaction", *Anal. Biochem.* **95**, 351–358.

Romero, F.I., Amengual, O., Atsumi, T., Khamashta, M.A., Tinahones, F.J. and Hughes, G.R.V. (1998) "Arterial disease in lupus and secondary antiphospholipid syndrome: association with anti-$\beta_2$-glycoprotein I antibodies but not with antibodies against oxidized low-density lipoprotein", *Br. J. Rheumatol.* **37**, 883–888.

Ross, R. (1999) "Atherosclerosis: an inflammatory disease", *N. Engl. J. Med.* **340**, 115–126.

Roubey, R.A.S. (1994) "Autoantibodies to phospholipid-binding plasma proteins: a new view of lupus anticoagulants and other 'antiphospholipid' antibodies", *Blood* **84**, 2858–2867.

Salonen, J.T., Yla-Herttuala, S., Yamamoto, R., *et al.* (1992) "Autoantibodies against oxidized LDL and progression of carotid atherosclerosis", *Lancet* **339**, 883–887.

Senti, M., Tomas, M., Vila, J., *et al.* (2001) "Relationship of age related myocardial infarction risk and Gln/Arg 192 variants of the human paraoxonase 1 gene. The REGICOR study", *Atherosclerosis* **156**, 443–449.

Sheng, Y., Kandiah, D.A. and Krilis, S.A. (1998) "$\beta_2$-glycoprotein I: target antigen for 'antiphospholipid' antibodies. Immunological and molecular aspects", *Lupus* **7**, S5–S9.

Steinberg, D. (1997) "Low density lipoprotein oxidation and its pathobiological significance", *J. Biol. Chem.* **272**, 20963–20966.

Steinberg, D. (2002) "Atherogenesis in perspective: hypercholesterolemia and inflammation as partners in crime", *Nature Med.* **8**, 1211–1217.

Tan, E.M., Cohen, A.S., Fries, J.F., *et al.* (1982) "The 1982 revised criteria for the classification of systemic lupus erythematosus", *Arthritis Rheum.* **25**, 1271–1277.

Tinahones, F.J., Cuadrado, M.J., Khamashta, M.A., *et al.* (1998) "Lack of cross-reaction between antibodies to $\beta_2$-glycoprotein-I and oxidized low-density lipoprotein in patients with antiphospholipid syndrome", *Br. J. Rheumatol.* **37**, 746–749.

Vaarala, O. (1996) "Antiphospholipid antibodies and atherosclerosis", *Lupus* **5**, 442–447.

Vaarala, O. (1998) "Antiphospholipid antibodies in myocardial infarction", *Lupus* **7**, S132–S134.

Vaarala, O., Alfthan, G., Jauhiainen, M., Leirisalo-Repo, M., Aho, K. and Palosuo, T. (1993) "Crossreaction between antibodies to oxidized low density lipoprotein and to cardiolipin in systemic lupus erythematosus", *Lancet* **341**, 923–925.

Van Doornum, S., McColl, G. and Wicks, I.P. (2002) "Accelerated atherosclerosis. An extraarticular feature of Rheumatoid Arthritis?", *Arthritis Rheum.* **46**, 862–873.

Virella, G., Atchley, D.H., Koskinen, S., Zheng, D. and Lopes-Virella, M. (2002) "Pro-atherogenic and pro-inflammatory properties of immune complexes prepared with purified human oxLDL antibodies and human oxLDL", *Clin. Immunol.* **105**, 81–92.

Ward, M.M. (1999) "Premature morbidity from cardiovascular and cerebrovascular diseases in women with systemic lupus erythematosus", *Arthritis Rheum.* **42**, 338–346.

Wilson, W.A., Gharavi, A.E., *et al.* (1999) "International consensus statement on preliminary classification criteria for definite antiphospholipid syndrome: report of an international workshop", *Arthritis Rheum.* **42**, 1309–1311.

Yla-Herttuala, S., Palinski, W., Rosenfeld, M.E., *et al.* (1989) "Evidence for the presence of oxidatively modified low density lipoprotein in atherosclerotic lesions of rabbit and man", *J. Clin. Investig.* **85**, 1086–1095.

Zhao, D., Ogawa, H., Wang, X., *et al.* (2001) "Oxidized low-density lipoprotein and autoimmune antibodies in patients with antiphospholipid syndrome with a history of thrombosis", *Am. J. Clin. Pathol.* **116**, 760–767.

